# Inactive disease and remission rates after intraarticular steroids as initial therapy for juvenile idiopathic arhtritis (JIA)

**DOI:** 10.1186/1546-0096-9-S1-P207

**Published:** 2011-09-14

**Authors:** JO Sato, TAP Fernandes, CB Nascimento, RG Brito, C Saad-Magalhães

**Affiliations:** 1 Pediatric Rheumatology Unit, Botucatu Medical School, São Paulo State University (UNESP), Brazil

## Objective

Describe rates of Inactive Disease (ID) and remission on (CRM) and off medication (CR) after intraarticular steroids (IAS) as initial therapy for JIA.

## Methods

Frequency of ID, CRM and CR were calculated and estimated by survival curves for all subjects with initial treatment of IAS until additional therapy with DMARDs or biologics was added.

## Results

A review of 110 IAS sessions (246 treated joints) in 72 subjects was carried out. Median (IQR) follow-up duration was 4.3 years (2.7 – 6.1). JIA (ILAR) categories were: persistent (55.6%) and extended oligoarthritis (27.8%), enthesitis related arthritis (8.3%), undifferentiated (6.9%) and psoriasic (1.4%). Knee (s) were treated in 47.3%, both knees and ankles in 38.2%, and only ankle (s) in 14.5%. Triamcinolone hexacetonide was used in 86.1%. ID rates after the first IAS (n= 72) was 55.6%, at median (IQR) 2.4 months (1.1 – 6.6). After the first IAS session, CRM and CR were observed in 25% and 22.2%, respectively. After the second (n=22), ID, CRM and CR occurred in 45.5%, 13.6% and 9.1%, respectively, and after the third (n=12), 16.7%, 0% and 8.3%, respectively. Overall outcome was: CR in 23.6%, CRM in 4.2%, 18.1% lost follow-up, 34.7% added DMARD and 4.2% DMARD plus biologic. Median survival for inactive disease, CRM and CR were 6.2, 11.5 and 21.6 months.

## Conclusions

Inactive disease state occurred in about 50% and CR in 23.6% of patients. Effectiveness of IAS tends to decreased with repeated injections, and this might be considered to step up treatment.

